# Interocular Grouping in Perceptual Rivalry Localized with fMRI

**DOI:** 10.1007/s10548-021-00834-4

**Published:** 2021-04-19

**Authors:** Athena Buckthought, Lisa E. Kirsch, Jeremy D. Fesi, Janine D. Mendola

**Affiliations:** 1 Department of Ophthalmology, McGill University, Montreal, QC H9G 1A4 Canada; 2 School of Physical and Occupational Therapy, McGill University, Montreal, QC H9G 1A4 Canada; 3 Montreal General Hospital, 1650 Cedar Avenue, Rm. L7-120, Montreal, QC H9G 1A4 Canada

**Keywords:** Binocular, Monocular, Flicker-and-swap, Lateral occipital, Gestalt, Illusory contours

## Abstract

Bistable perception refers to a broad class of dynamically alternating visual illusions that result from ambiguous images. These illusions provide a powerful method to study the mechanisms that determine how visual input is integrated over space and time. Binocular rivalry occurs when subjects view different images in each eye, and a similar experience called stimulus rivalry occurs even when the left and right images are exchanged at a fast rate. Many previous studies have identified with fMRI a network of cortical regions that are recruited during binocular rivalry, relative to non-rivalrous control conditions (termed replay) that use physically changing stimuli to mimic rivalry. However, we show here for the first time that additional cortical areas are activated when subjects experience rivalry with interocular grouping. When interocular grouping occurs, activation levels broadly increase, with a slight shift towards right hemisphere lateralization. Moreover, direct comparison of binocular rivalry with and without grouping highlights strong focused activity in the intraparietal sulcus and lateral occipital areas, such as right-sided retinotopic visual areas LO1 and IP2, as well as activity in left-sided visual areas LO1, and IP0-IP2. The equivalent analyses for comparable stimulus (eye-swap) rivalry showed very similar results; the main difference is greater recruitment of the right superior parietal cortex for binocular rivalry, as previously reported. Thus, we found minimal interaction between the novel networks isolated here for interocular grouping, and those previously attributed to stimulus and binocular rivalry. We conclude that spatial integration (i.e,. image grouping/segmentation) is a key function of lateral occipital/intraparietal cortex that acts similarly on competing binocular stimulus representations, regardless of fast monocular changes.

## Introduction

The ability to experience more than one percept while the physical stimulus remains constant is called multistable perception. This phenomenon occurs when visual input is ambiguous and compatible with more than one mutually exclusive interpretation (Sterzer et al. 2009). Paradigms such as binocular rivalry and stimulus rivalry are types of multistable perception that have been widely investigated (e.g., Logothetis et al. 1996; Lee and Blake 1999; Van Boxtel et al. 2008; Buckthought et al. 2015; Petruk et al. 2018). By examining perceptual rivalry, insight can be gained into the neural mechanisms involved in resolving visual ambiguity, fragmentation, and conflict in general (Silver and Logothetis 2004; Sterzer et al. 2009). In particular, rivalry with manipulation of *interocular grouping* is a valuable tool for investigating how the visual system integrates visual elements across eyes, and space, to formulate a coherent perceptual experience, commonly known as visuospatial integration.

Interocular grouping refers to coherent percepts composed of parts that originate from different eyes. The simplest paradigm consists of dichoptic stimuli (different for each eye) presented to the observer that can form coherent global shapes but are broken up into patches that are distributed between both eyes (Diaz-Caneja 1928 cited in Alais et al. 2000). In their seminal paper, Kovacs et al. (1996) used patches of highly complex chromatic stimuli, made up of a monkey face and a series of words on a colored background. Observers reported perceiving the entire face or all the words, with the two percepts alternating over time. Hence, combining interocular grouping with rivalry results in pattern coherency due to the recombination of each eye’s monocular, non-uniform input stimuli (Knapen et al. 2007). Although local mechanisms cannot be entirely ruled out (e.g., Lee and Blake 2004), this phenomenon suggests that perceptual dominance of a coherent image is largely based upon a global process of spatial integration that spans long-range distances of several degrees of visual angle. (Suzuki and Grabowecky 2002; Jacot-Guillarmod et al. 2017). Moreover, it has been shown that periods of dominance for multiple rivalrous regions of an image are more likely to co-vary synchronously when they share common features such as colour or motion (Alais and Blake 1998, 1999; Kovacs et al. 1996; Stuit et al. 2011). There are also other Gestalt cues that have been shown to reinforce the grouping process, such as good continuation and common fate (Alais and Blake 1999).

In addition to spatial integration, visual features need to be integrated in time. So-called stimulus rivalry is a variation of binocular rivalry that addresses this temporal domain. Also referred to as the “flicker and eye-swap” technique, dichoptic presentation of dissimilar patterns (e.g., orthogonal gratings), are periodically exchanged between the eyes at a fast rate, (Logothetis et al. 1996; Lee and Blake 1999; see also Van Boxtel et al. 2008; Buckthought et al. 2015). Again, the perception alternates between two patterns. Since a single perceptual dominance phase lasts for multiple stimulus exchanges between the eyes, the rivalrous competition cannot be based solely on the eye of origin. The issue of whether stimulus and binocular rivalry paradigms actually engage the same neural mechanism is currently still up for debate. TMS applied to early cortical visual areas (V1 and surrounding) disrupts normal perceptual alternations during binocular rivalry but not stimulus rivalry (Pearson et al. 2007). Nevertheless, recent fMRI and MEG studies found a similar pattern of activation for both conditions, but with binocular rivalry producing greater activity (Buckthought et al. 2015; Petruk et al. 2018).

We hypothesized that fMRI examination of interocular grouping with *both* binocular rivalry *and* stimulus rivalry might shed light on how the visual system integrates visual features, both spatially and temporally. In addition, we aimed to re-examine the mechanistic commonalities between binocular and stimulus rivalry, as it pertains to interactions with interocular grouping (e.g. Lee and Blake 2004). This type of comprehensive fMRI comparison has not been reported previously. To our knowledge, only one study exists that attempted to examine similar interactions, but using EEG (see ‘Discussion’) (Sutoyo and Srinivasan 2009).

Given these complex paradigms, the critical neural substrates of rivalry *with* interocular grouping seem likely to include extrastriate regions with 1. large receptive fields, and 2. object selectivity, such as the lateral occipital complex (LOC) (Grill-Spector and Kanwisher 2001). It has been established that the LOC has stronger activation for whole formed objects whether familiar or not, than with images with no evident configuration (Malach et al. 1995). Furthermore, it is also activated by object fragments (Grill-Spector et al. 1998). The LOC is also strongly engaged in object shape encoding, regardless of the visual features or cues used to characterize the object (Kourtzi and Kanwisher 2001).

Finally, perceptual grouping is part of a larger “spatial binding problem” which refers to how the visual system synthesizes the visual world into a coherent experience, rather than seeing a disjointed world, with wrongly combined visual features (Treisman 1998). Spatial attention is necessary for this ability especially when there are several objects presented simultaneously (e.g., Shafritz et al. 2002). These functions have been repeatedly associated with the parietal cortex (Shafritz et al. 2002; Robertson et al. 1988; Seymour et al. 2008; Friedman-Hill et al. 1995), and thus may be required for interocular grouping. For example, by using a bistable stimulus which leads to alternations between local features versus a grouped illusory Gestalt percept, Zaretskaya et al. (2013) showed that fMRI activity in the right posterior parietal cortex, superior parietal lobe, and the right anterior intraparietal sulcus was linked to the unified grouped percept, as compared to the percept of just its parts.

In the current study, we aimed to determine the neural correlates of perceptual grouping with rivalry paradigms using psychophysics and fMRI. We created stimuli for binocular rivalry and stimulus rivalry that were identical (except for eye swapping), and that resulted in comparable bistabilty, with similar alternation rates. We applied standard fMRI methods while relating our results to *retinotopic* visual areas defined individually in our subjects or via a probabilistic atlas (Wang et al. 2015; Rosenke et al. 2018). In sum, the objectives of this study are: (1) to investigate whether interocular grouping during rivalry changes the intensity of the BOLD signal, and/or requires recruitment of additional neural networks, and (2) to compare perceptual grouping during binocular and stimulus rivalry.

## Methods

### Subjects

Two authors (AB and LK) and four subjects who were naïve as to the hypotheses of the study participated in all experiments. The subjects (which included five women) were university students or postdoctoral fellows. All were right-handed and had normal or corrected-to-normal acuity and stereoacuity thresholds better than 30 s arc, measured using the Titmus stereo test (Stereo Optical Co., Chicago, IL). The subjects provided informed consent and were remunerated for their time. The experiments were approved by the Research Ethics Board (REB) of the McGill University Health Centre (Protocol NEU-08-03).

### Display

All stimuli were presented on a MacBook Pro Laptop (Intel Core 2 Duo) Macintosh computer with 1024 × 768 resolution, 120 Hz refresh rate with 8 bit/pixel greyscale. Stimuli were generated and displayed using Matlab (2008b) and Psychtoolbox Version 3 (PTB-3) software. A Matrox (Dual Head 2Go Analogue) splitter graphics card was used to create a dichoptic display. Two LCD (InFocus LP 540) projectors and linear polarizers were used for dichoptic projection. From the projectors, the stimuli were back-projected onto a screen, which was 134 cm away from the subject. Projectors were calibrated using gamma correction. Recalibration was performed whenever there was an observed difference in luminance between the projectors. Stimuli had a mean luminance of 32 cd/m^2^ and peak luminance of 64 cd/m^2^. Subjects wore linear polarizers with complementary polarization of the glasses. During the psychophysical testing, which preceded the fMRI testing, each subject was given two different types of polarized lens glasses in order to counterbalance for which projector sent an image to each eye during testing. For fMRI, one set of glasses was used. Subjects wore their respective prescription lenses underneath the polarized glasses if so required. The same display setup was used both for psychophysics and fMRI testing sessions, with the same stimulus sizes and viewing distances. During fMRI testing, the screen was placed at the rear end of the MR scanner bore and subjects viewed stimuli through a mirror attached to the head coil. Prior to their fMRI session, all subjects had a brief practice session.

### Stimulus Conditions

**Fig. 1:**
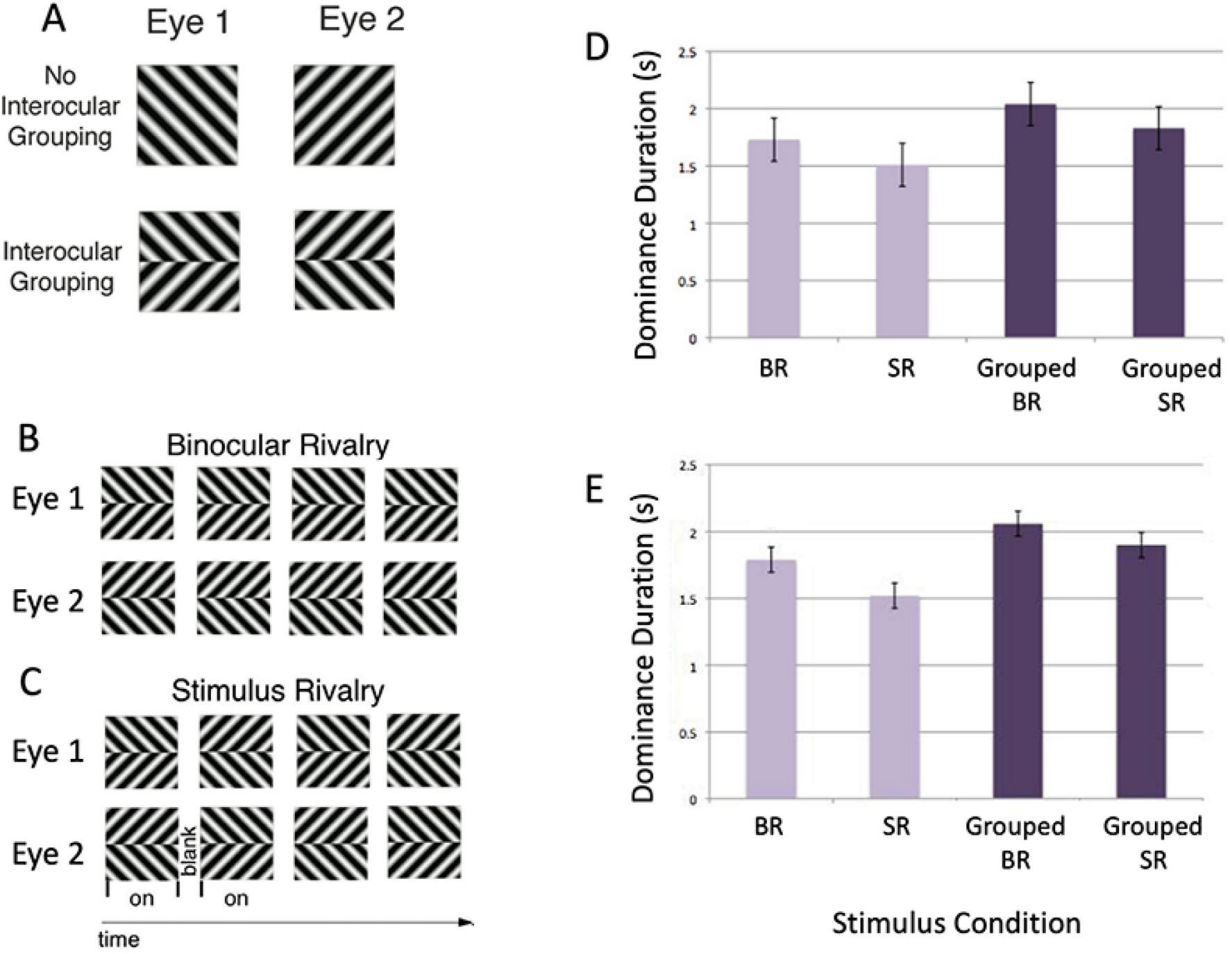
Stimuli and behavioral results. **a** Binocular rivalry stimuli were viewed dichoptically and, in the case of interocular grouping, were comprised of two parts in each eye that combined perceptually. **b** and **c** Stimulus rivalry conditions were identical to binocular rivalry except for stimulus exchange between eyes at a rate of 6.7 Hz (grouped conditions shown). **d** and **e** All four stimulus conditions produced rivalry with comparable alternations rates in pre-fMRI testing (**d**) and during fMRI scanning (**e**). The differences between conditions were not significant

There were four rivalry conditions compared during the psychophysical testing: binocular and stimulus rivalry with and without perceptual grouping (Fig. 1). Each block of rivalry lasted 90 s. All of the conditions were comprised of achromatic orthogonal sine wave gratings ± 45 deg (left or right oriented) with a spatial frequency of 1.4 cycles per degree at 80% Michelson contrast. In order to best match the temporal transients that occur in stimulus rivalry, all conditions periodically blanked using the same 67 ms period, generating a flicker of 6.67 Hz (methods similar to Buckthought et al. 2015). In other words, binocular and stimulus rivalry conditions had the same temporal frequencies. For the psychophysics that preceded the fMRI, the stimulus on period was either 100 or 83 ms. These two stimulus on period rates were tested to determine the optimal parameters for equalizing the alternation rates across conditions. Therefore, version 1 had a 100 ms stimulus on period followed by a 67 ms blank period, making a 167 ms total pattern of repetition. Version 2 had a stimulus on period of 83 ms followed by a 67 ms blank period, making a 150 ms total pattern of repetition. For all conditions, subjects perceived either a predominantly left oriented grating or predominantly right oriented grating during a given alternation. Predominance is defined as perceiving a particular grating orientation over at least two thirds of the image. See Fig. 1 for images of the stimuli.

#### Binocular Rivalry

Through the polarized lenses, each eye is shown an achromatic ± 45 deg (left or right) oriented grating. Each eye is always shown one of two orientations for the duration of the trial, with a blank period of 67 ms and on period of 100 or 83 ms.

#### Stimulus Rivalry

The stimulus is similar to binocular rivalry except that the oriented gratings were continually exchanged (“swapped”) between the eyes either every 167 ms (parameter 1) or 150 ms (parameter 2) to induce stimulus rivalry. For fMRI, only the 83 ms stimulus on period was used leading to an exchange rate of 150 cycles and a flicker of 6.67 Hz.

#### Grouped Binocular Rivalry

This stimulus is comprised of an upper and lower half that differs in orientation. The top half of one eye’s stimulus is grouped with the bottom half of the other eye’s stimulus for a global percept. The other parameters are the same as binocular rivalry.

#### Grouped Stimulus Rivalry

This condition is identical to the grouped binocular rivalry condition with the addition of left and right stimulus exchange every 167 or 150 ms. There is always a blank period inserted between each interocular exchange of 67 ms. Again, for fMRI, only the 150 ms condition was used.

### Psychophysical Task and Procedure

For *key press task 1*: Subjects were instructed to press the 1 key on the laptop keyboard when they perceived a predominantly left oriented grating or if a composite (mixed percept) was perceived. Subjects pressed the 2 key when they perceived a predominantly right oriented grating. For *key press task 2*: Subjects pressed the 1 key when subjects a predominantly left oriented grating was perceived and the 2 key was pressed when a predominantly right oriented grating was perceived or if a composite was perceived. This was done in order to deduce whether subjects had a dominant eye and to measure the time that a composite was perceived. Subjects practiced these tasks until they felt comfortable performing the task for all conditions. Alternation rates were measured for each stimulus condition with key presses made continuously for a period of 90 s. Each testing session comprised of four sets of the four stimulus conditions, randomized (4 × 4 = 16 trials of 90 s per stimulus condition). All stimulus conditions were randomized between subjects as well. Each set also had a certain pair of polarized glasses (glasses 1 or 2). Each pair of glasses allowed a specific projector to be viewed by a specific eye only, so that projector was counterbalanced.

### Functional Magnetic Resonance Imaging

Data Acquisition: Six subjects were scanned on a Siemens 3T MRI machine at the McConnell Brain Imaging Centre at the Montreal Neurological Institute. The TIM Trio MR scanner was equipped with a 32 channel head coil. Functional whole brain images were acquired using a T2*-weighted gradient echo, echo-planar imaging sequence (38 slices, repetition time (TR) 2500 ms, echo time (TE) 30 ms, FOV 224, voxel size 3 × 3 × 3 mm). For each subject, anatomical images were acquired by using a T1-weighted magnetization-prepared rapid gradient-echo (MP-RAGE) sequence optimized for contrast between grey and white matter (176 slices, inversion time (TI) 900 ms, repetition time (TR) 2300 ms, echo time (TE) 2.98 ms, FOV 256, voxel size 1 × 1 × 1 mm).

A blocked design comprised of 30-s epochs was used. There were eight scans, 11 blocks per scan, each one being 5 min and 30 s. Subjects were given a button box in order to make key presses. Subjects were asked to perform key press task 1 for the first four scans and key press task 2 for the last four scans. Each scan was divided into two halves. The first half contained a randomized set of the *four rivalrous stimulus conditions* previously described as well as a *blank baseline* condition. The last half of the scan was comprised of *four replay conditions* as well as two other control conditions, which will not be discussed. For the non-rivalrous replay conditions, the exact timing of key presses and dominance durations calculated in the first half of the scan were stored and used to mimic the perceptual experience of rivalry by alternating stimulus orientation while subjects view stimuli *matched* in each eye. We did not attempt to show composite or ‘mixed’ percepts during replay. During the replay conditions, subjects were asked to press the 1 or 2 key depending on which orientation they were viewing and depending on the key press type for that particular active scan session. By later subtracting the replay conditions from their corresponding “task” condition, neural activity from the sensorimotor elements of performing the task could be removed from the analysis isolating rivalry per se. For all conditions, a fixation cross was presented for 0.8 s at the beginning of each block. Between each block, a brief blank period of 0.5 s occurred, to alert the subjects of a change in condition.

#### Retinotopic Mapping and Localization of MT+ and LOC

Retinotopic mapping was performed in a separate scan session (e.g., Buckthought et al. 2015). Four runs were performed for each subject. Stimuli consisted of two types of high contrast, chromatic, flickering checkerboard patterns. A rotating wedge stimulus swept through polar angles (for polar mapping; clockwise and counter-clockwise) and an expanding and contracting ring image was used to map eccentricity (fovea to periphery). Both stimuli increased in size in the periphery to compensate for cortical magnification (Sereno et al. 1995). The eccentricity stimuli traversed space using a logarithmic transformation. Polar mapping runs were 8 cycles of the full field rotating wedge, lasting 512 s. Eccentricity mapping were 8 cycles of expanding & contracting rings, lasting 512 s. A central fixation marker was present, and subjects were asked to perform a task by monitoring the orientation of the marker. This was done to maintain central fixation. The retinotopic scans were used to define so-called *foveal* regions of interest in V1, V2 and V3, defined as the region of occipital pole activated in the central 5.3 degrees of visual angle. Area V3A was also defined using these scans. Two runs were also performed for area MT+ localization, consisting of 8 epochs of 16 s of low contrast stationary rings or moving rings (Tootell et al. 1995). Lastly, subjects performed two runs of LOC localizer scans, which consisted of six blocks of photographs of objects alternating with six blocks of scrambled objects as well as a blank baseline condition. Each block was 20 s long and 40 images were shown per block. Regions of interest were defined using a t-test (p < 10^–4^ FDR) that detected regions that were significantly more activated with intact versus scrambled objects. Stimuli were obtained from the Kanwisher lab (Grill-Spector et al. 1998).

#### Additional Regions of Interest

In addition, four other regions of interest were defined as areas of contiguous voxels using previously performed statistical subtractions (p < 0.01 FDR for all cases) (Buckthought et al. 2015) that isolated prominent regions in the binocular rivalry network. The superior parietal area (SP) was defined bilaterally as an area more strongly activated by passive binocular rivalry than a non-rivalrous control condition. The temporoparietal junction (TPJ) was also defined in the right hemisphere as an area in the inferior parietal lobe, which was more strongly activated by the passive (no-task) binocular rivalry condition than the non-rivalrous condition. The passive rivalry conditions were far more effective in localizing the SP and TPJ regions. However, the ventral temporal area (VT) could only be defined well bilaterally as a region more strongly activated during the active-task binocular rivalry condition than an active nonrivalrous control condition. Finally, the lateral occipital region (LOR) was defined bilaterally as an area more strongly activated by grouped binocular rivalry condition than ungrouped binocular rivalry using the data collected in the current study. Based on the topography of that subtraction in the average map of all subjects, this area was parceled into a non-contiguous ventral and dorsal area, named LOR-V and LOR-D respectively. It should be noted that using this region to query the same subtraction (Grouped BR-BR) is not independent. However, we include this ROI as a helpful benchmark, not only for other subtractions, but also for other ROIs, as explained in Results. Finally, all four of these functionally defined ROIs were defined based on the average map of all subjects, and then morphed back onto the individual subject’s data to calculate estimates of BOLD percent signal change.

#### Data Analysis

BrainVoyager QX analysis package, version 2.1.2.1545 (Brain Innovations, Maastricht, The Netherlands) was used for all functional data analyses as well as for the creation of inflated and flattened cortical representations. The anatomical and functional scans were analyzed in BrainVoyager using the standard sequence in this software package, described as follows. The anatomical scans were used to create surface reconstructions of each subject’s cerebral cortex. The computed cortical surface representation was inflated and then flattened. Each subject’s reconstructed folded cortical representation was normalized to spherical coordinate space and aligned to a target brain (chosen as an individual subject) using cortex-based alignment (Goebel et al. 2004). The cortex-based alignment was performed in order to obtain a good match between corresponding brain regions for the group-level statistical data analysis.

Before analysis of the functional scans, the first two volumes of every scan were discarded. All functional images were preprocessed as follows: (1) 3D motion correction; (2) slice timing correction; (3) linear trend removal using a high-pass filter; (4) transformation of the functional data into Talairach coordinate space (Talairach and Tournoux 1988); and (5) coregistration to anatomical images. A voxel-by-voxel, mixed effects summary statisitics general linear model (GLM) was used for analysis that explicitly takes the variability between subjects into account. The functional results were then viewed on an individual’s cortical surface, producing maps of statistical significance (t-tests with a false discovery rate of p < 0.05), which were spatially smoothed. In addition, we separately analyzed the BOLD signal changes within regions of interest (described above), using this mixed effects GLM analysis with a threshold of p < 0.005, to account for multiple comparisons.

The retinotopic atlas of Wang et al. (2015) was visualized on a BrainVoyager average surface, BV20, based on 20 brains (comparable to fsaverage in FreeSurfer). This had been previously transferred from FreeSurfer (Rosenke et al. 2018). Within BrainVoyager, “align each entry to target sphere” option with Cortex Based Alignment was used, choosing the BV20 as the target sphere. This made it possible to display the atlas patches of interest (POIs) on either the BV20 surface or the surface of an individual subject. This atlas allowed definition of several additional areas beyond V1-3 and V3A, that were mapped in our subjects. In addition, these independently defined retinotopic areas are thought to be meaningful cortical units, thus complementary to the functionally defined regions of interest described in section ‘Additional Regions of Interest’.

## Results

### Psychophysics Results

In order to optimize the comparison of binocular and stimulus rivalry, two different sets of stimulus cycle periods were tested. As mentioned previously, these were 167 ms or 150 ms. Mean dominance durations were calculated for each stimulus condition for both cycle periods. The optimal cycle period, 150 ms, showed the smallest differences between the means of the four stimulus conditions, and was thus was chosen for fMRI. Furthermore, this was also the condition that subjects self-reported they could perform the most optimally. In this way, we were successful in matching the alternation rates for binocular and stimulus rivalry (Fig. 1d, e).

For the psychophysics, a two-way ANOVA confirmed that there were no significant differences or interactions between the mean dominance durations for the binocular and stimulus rivalry conditions or the grouped and ungrouped conditions (rivalry type F(1, 20) = 0.40, p = 0.53; grouped or not F(1, 20) = 1.49, p = 0.24; interactions F(1, 20) = 0.08, p = 0.78). The mean dominance durations for all subjects were 1.78 s for binocular rivalry versus 1.69 s for stimulus rivalry. This corresponds to alternation rates of 0.64 alternation/s for binocular and 0.66 for stimulus rivalry (*t*(5) =  − 0.57, *p* > 0.05). For the key press data collected during the fMRI sessions, we note that less data were collected overall, and were collected over a greater number of shorter epochs. Nevertheless, calculated alternation rates were similar, 0.58 and 0.70 alternation/s, respectively. The corresponding mean dominance durations were 1.77 s and 1.52 s, respectively. A two-way ANOVA confirmed that there were no differences between grouped and ungrouped conditions (F(1,5) = 5.183, p = 0.07), while the differences between binocular and stimulus rivalry conditions were statistically significant (F(1,5) = 10.232, p = 0.024), but the interaction was not significant (F(1,5) = 0.193, p = 0.678). Post-hoc paired t-tests were performed comparing the stimulus conditions and none of these differences were statistically significant when corrected for multiple comparisons with Bonferroni correction (BR vs. grouped BR, t(5) = 3.05, p > 0.0125, BR vs. SR, t(5) = 2.07, p > 0.0125, grouped BR vs. grouped SR, t(5) = 1.534, p > 0.0125, grouped SR vs. SR, t(5) = 1.443, p > 0.0.125). With regard to mixed percepts, the mean percentage was 10% for binocular rivalry and 14% for stimulus rivalry, and there was no significant difference between the conditions, *t*(5) = 0.95, *p* > 0.05.

### fMRI Results

#### Comparison of Binocular Rivalry to Replay, Both Ungrouped and Grouped

**Fig. 2:**
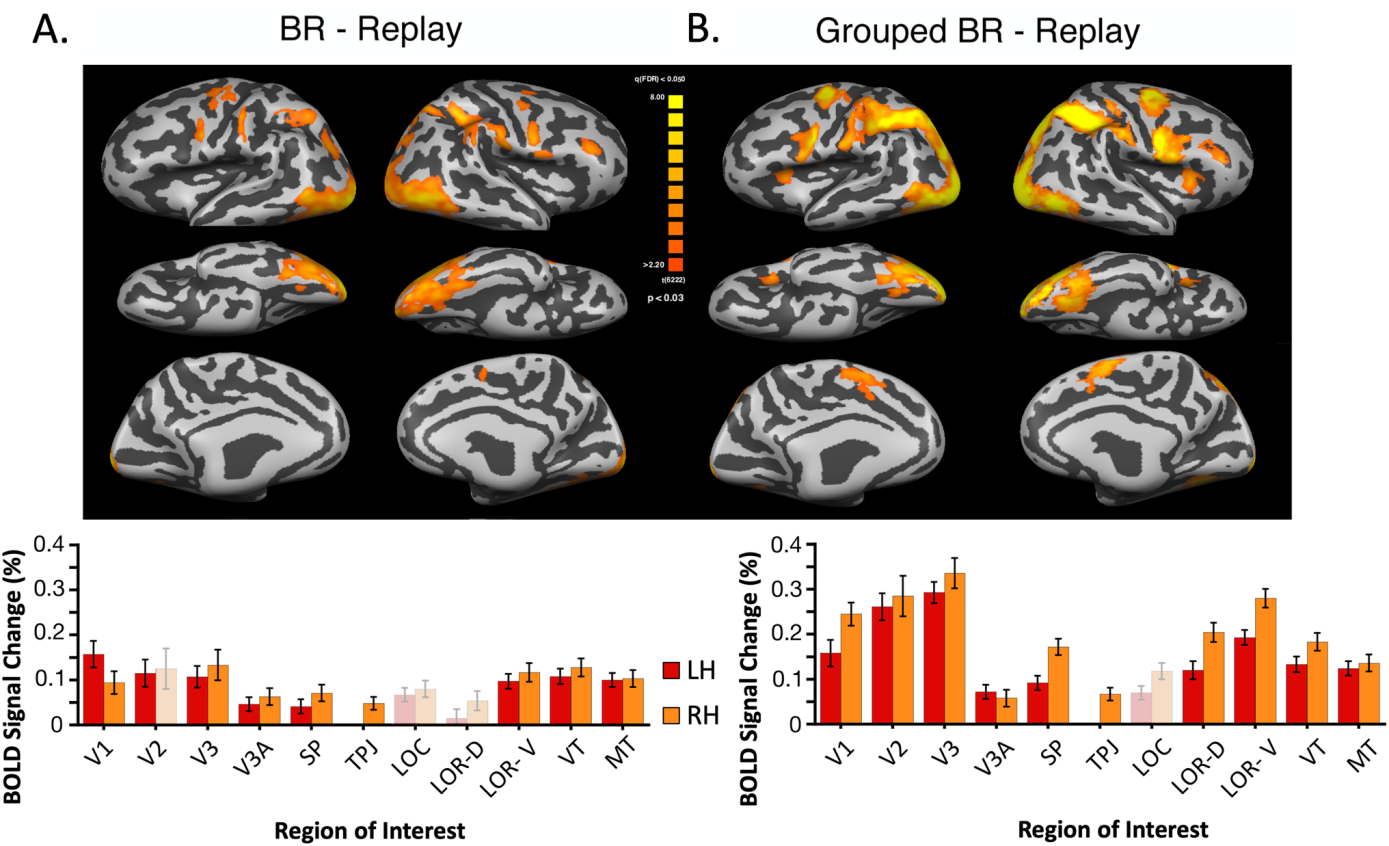
Comparison of binocular rivalry with and without interocular grouping. **a** Average brain maps for the binocular rivalry task compared to the replay task (N = 6). Bar graphs below the maps indicate the regions of interest (ROI) where the differences between tasks were significant. **b** Average brain maps for binocular rivalry with grouping task compared to replay task. With false-discovery-rate (FDR) correction for multiple comparisons in Brain Voyager, the exact p-values vary slightly for each hemisphere in each subtraction. The values ranged from p < 0.014 to 0.028. Bar graphs below the maps show the ROIs where the % BOLD differences between tasks were significant with bright color; non-significant regions shown with pale color. Both bar graphs show results for right and left hemisphere separately; error bars plot SED

It is useful to first consider the neural network that is activated during classic binocular rivalry, without manipulation of interocular grouping. By subtracting the replay condition from the rivalry condition, the aim is to remove the sensorimotor responses and isolate the influence of ambiguous and competitive rivalry. This results in the brain maps showing areas that are active during binocular rivalry per se (Fig. 2a). In comparison, the pattern of activation for binocular rivalry with grouping, compared to its replay, is similar, however grouped binocular rivalry clearly evokes greater activity overall (Fig. 2b). These group results were well supported for the six individual subjects, the only exceptions were very rarely to show no activation above threshold in one or both hemispheres. For BR—Replay this occurred in 4/12 hemispheres, and in 1/12 hemispheres for BR Grouping—Replay. This pattern was consistent with greater overall activation when grouping demands were present.

This data can also be visualized for each ROI in the bar graphs below the brain maps. For these graphs, bars are brightly colored for the ROIs in which the % BOLD change is significantly greater than zero. Data that failed to reach significance is plotted in pale color. For rivalry with grouping, there is higher BOLD signal change in retinotopic areas, V1, V2 and V3 (t = 5.31–12.1, p < 0.001). Also, as we would expect from the LOR definition, there is a high level of activation in regions of interest LOR-D and LOR-V (t = 6.02–12.35, p < 0.001). As mentioned later, these areas may be responsible for global shape formation and surface selection. In addition, region SP (t = 6.48–9.44, p < 0.001), especially in the right hemisphere, was highly active for grouped rivalry, which might point to increased spatial attention. There is also a small trend towards the right hemisphere for both grouped and ungrouped conditions, however it is more prominent in the grouped condition. Lastly, the functionally defined LOC region failed to reach significance for either condition, likely due to the large size and lack of selectivity of this region, as well as lack of overlap with the LOR region. This is considered in detail later, in Fig. 5.

#### Comparison of Grouped Binocular Rivalry and Ungrouped Binocular Rivalry

**Fig. 3:**
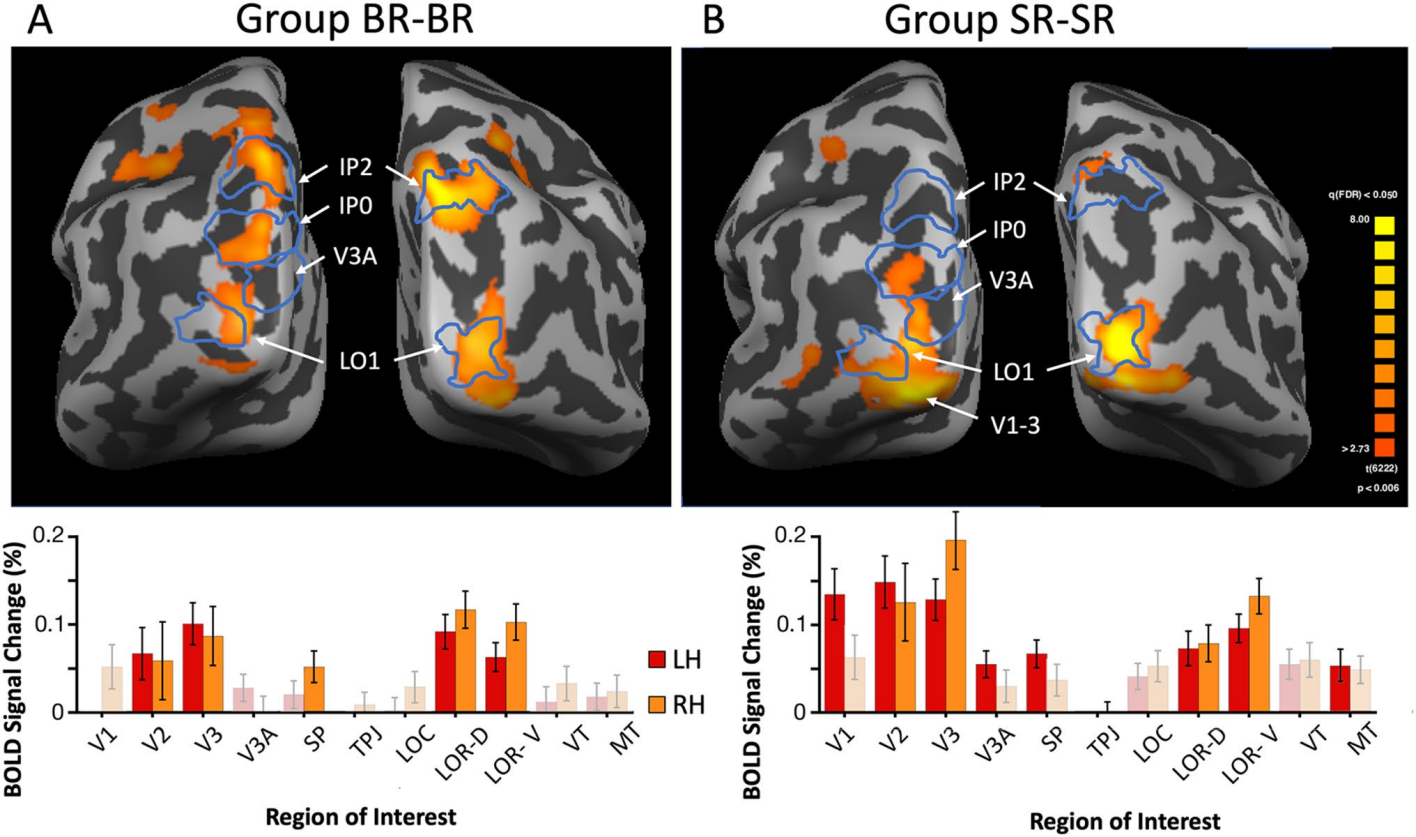
Comparison of binocular rivalry with grouping to stimulus rivalry with grouping. **a** Average brain maps for the binocular rivalry with grouping task compared to the binocular rivalry task (posterior brain view) (N = 6). **b** Average brain maps for the stimulus rivalry with grouping task compared to the stimulus rivalry task. With false-discovery-rate (FDR) correction for multiple comparisons in Brain Voyager, the exact p-values vary slightly for each hemisphere in each subtraction. The values ranged from p < 0.002–0.006. In both **a** and **b** blue outlines indicate the probabilistic location of cortical areas from the Wang atlas, along with area names in white. Bar graphs below the maps show the ROIs where the % BOLD differences between tasks were significant with bright color; non-significant regions shown with pale color. Both bar graphs show results for right and left hemisphere separately; error bars plot SED

The next step was a direct comparison between grouped and ungrouped binocular rivalry (Fig. 3a). These brain maps show stronger activation for grouped binocular rivalry along the lateral occipital aspect of the inflated cortex, both ventrally and dorsally. Again, this averaged result was supported in most individual subjects and hemispheres for Grouped BR–BR (and Grouped SR–SR) a lack of positive activation was seen in 2/12 (and 3/12) hemispheres. In addition, the bar graphs below confirm that the regions of interest V2, V3, SP as well as lateral occipital regions LOR-D and LOR-V (t = 3.4–5.7, p < 0.001) are significantly different for grouped binocular rivalry compared to ungrouped. Also shown on brain maps (blue lines) are the probabilistic retinotopic visual areas, including two intraparietal areas (IP2, IP0), V3A, and a lateral occipital area, LO1 (Wang et al. 2015). We have performed this registration in order to place our results in a common retinotopic coordinate space. In the right hemisphere, regions of maximal activation correspond very well to areas IP2 and LO1.

#### Comparison of Grouped Stimulus Rivalry and Ungrouped Stimulus Rivalry

Previous studies have already contrasted the neural substrates of classic binocular and stimulus rivalry in detail (Buckthought et al. 2015; Petruk et al. 2018), and we will consider this in the ‘Discussion’ section. Our current focus is to evaluate if the addition of interocular grouping affects stimulus rivalry differently than binocular rivalry. When we performed the analogous subtraction between grouped and ungrouped stimulus rivalry the results were quite similar overall to binocular rivalry with peaks of activation in lateral occipital regions LOR-D and LOR-V (Fig. 3b). Moreover, a separate formal analysis in BrainVoyager of the conjunction of Grouped BR–BR with Grouped SR–SR showed two significant regions, right-sided, LO1 and IP2, and thus emphasizes the consistent recruitment of those areas in all grouping conditions. However, more visual areas did show a significant increase in signal when grouped SR was directly compared to SR, including robust effects bilaterally in early visual areas, and ventral temporal and MT+ regions as well. This may be explained by the previously reported finding that compared to binocular rivalry, stimulus rivalry produces a reduced extent of BOLD activation overall. To better evaluate any differences between Fig. 3a and b, we performed a direct subtraction in the next section.

#### Comparison of Grouped Binocular Rivalry and Grouped Stimulus Rivalry

**Fig. 4:**
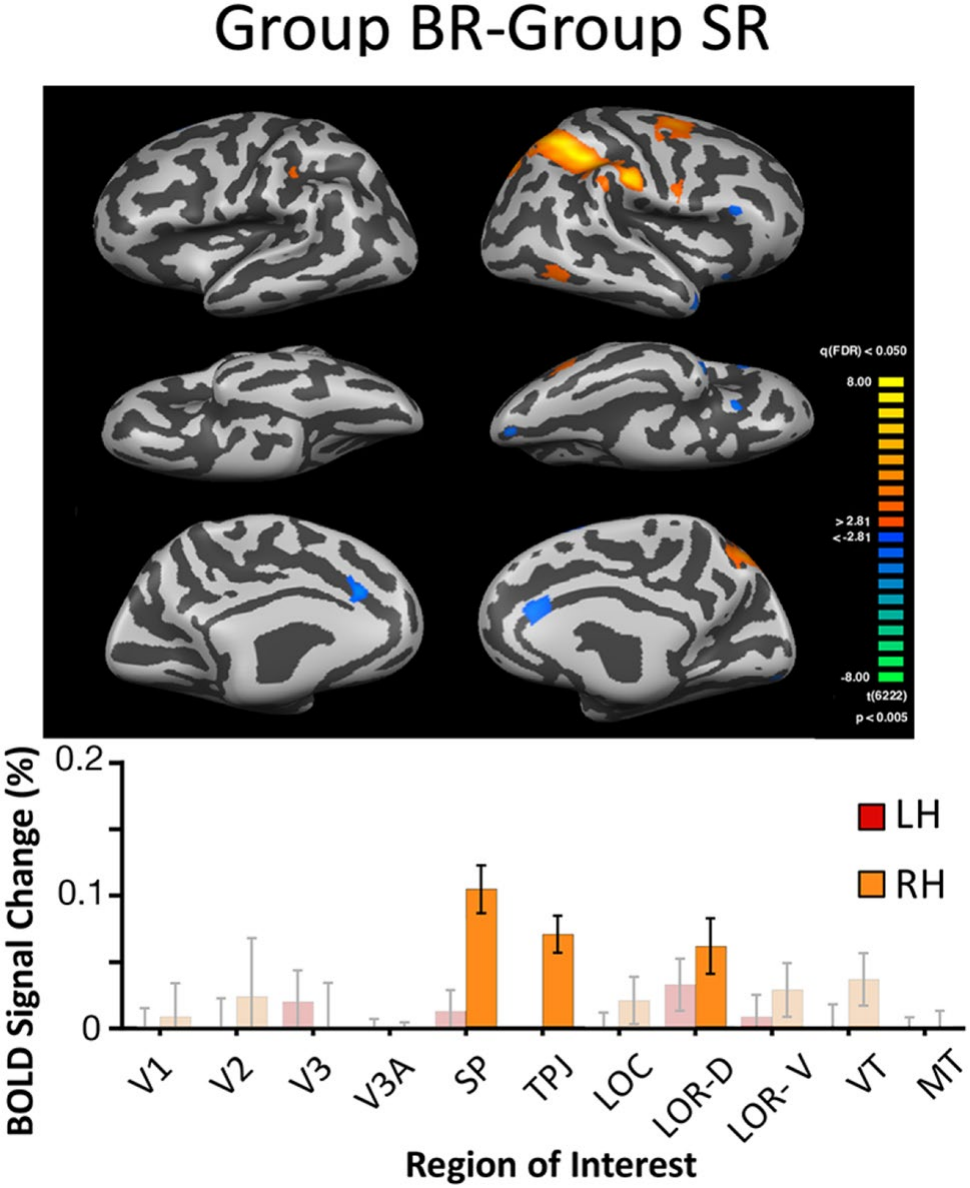
Direct comparison of grouped binocular and stimulus rivalry. Average brain maps for the binocular rivalry with grouping task compared to stimulus rivalry with grouping (N = 6). With false-discovery-rate (FDR) correction for multiple comparisons in Brain Voyager, the exact p-values vary slightly for each hemisphere. The values ranged from p < 0.001–0.005. Bar graphs below the maps show significant ROIs in bright colors, with highest signal change in region SP; error bars plot SED. A very strong right hemisphere lateralization is visible for both the maps and bars

**Fig. 5:**
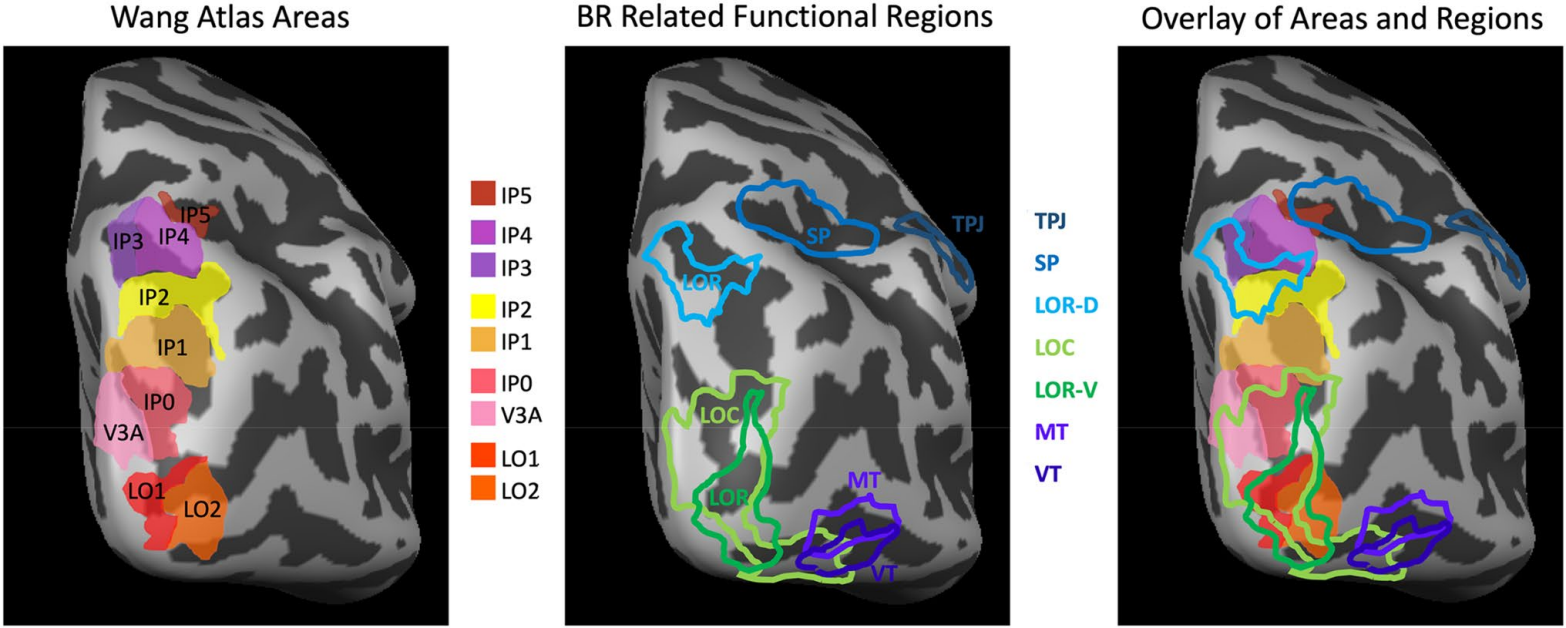
Topography of functional ROIs and probabilistic areas of Wang et al. (2015). Left panel shows inflated right hemisphere of one individual subject with selected areas from the Wang atlas, all shown filled in with warm colors. Center panel shows the same hemisphere with all of our functionally defined regions of interest outlined in cool colors. Right panel shows the registered overlay of both atlas areas and functionally defined regions. See adjacent text for description

While common areas of activation were broadly engaged by rivalry with interocular grouping regardless of the presence of left and right eye stimulus exchange, a direct comparison of grouped SR and grouped BR is needed to determine how stimulus exchange relates to image grouping (Fig. 4). When viewed on the brain maps, the largest difference is in the right parietal region (near visual areas IPS4 and IPS5 from the Wang atlas; see Fig. 5). Moreover, the bar graphs also suggest a bias in favor of BOLD signal in the right hemisphere, for regions of interest LOR-V, VT, LOR-D, TPJ, and SP. The bias towards the right hemisphere was strongest for area SP, and when tested statistically was highly significant (t(5) = 62.7;p < 0.001 This clear laterality effect is further considered in the ‘Discussion’. Finally, we also note in passing that there are small regions significantly more active for grouped SR than grouped BR, including anterior cingulate cortex. This activation is slight, but conceivably indicates some additional attentional or cognitive capacity may be required for grouped *stimulus* rivalry. Given that stimulus transients slightly reduce the clarity of stimulus rivalry, it might be speculated that adding a grouping manipulation requires additional cognitive control.

#### Topography of Functional ROIs and Probabilistic Areas

This final analysis was performed in order to better visualize and understand the extent of overlap between our multiple regions of interest and the probabilistic atlas of Wang et al. (2015). This atlas is very valuable for the estimation of the location of many retinotopically defined extrastriate visual areas that are difficult to activate sufficiently in individual subjects when scan time is limited. Figure 5 first shows nine of these areas, LO1, LO2, V3A, IP0, IP1, IP2, IP3, IP4, IP5 in warm colors. Our own functionally defined areas (TPJ, SP, LOC, LOR-D, LOR-V, MT, and VT) are outlined in cool colors. In particular, it can be appreciated that the LOC region (defined by more activity for objects than scrambled objects) is a large region encompassing both lateral occipital and posterior ventral temporal aspects of visual cortex. Our LOR-V region overlaps with LOC, as do small parts of MT and VT. Also, the right panel shows that LOC overlaps with the Wang atlas areas LO1, LO2, V3A, and IP0. We wish to emphasize that although the widely used LOC localizer does certainly overlap with LO1 (the area most consistently activated by rivalry with interocular grouping), the LOC is too large and non-selective to reach significance in the subtractions we report here. Finally, in the occipital-parietal aspect, it can be appreciated that our region LOR-D corresponds well to IP2 and IP3. Our SP region overlaps with IP5, while TPJ falls outside the atlas zone.

## Discussion

In these experiments, psychophysics and fMRI were used to investigate the neural correlates of interocular grouping. We found that rivalry with interocular grouping prominently recruits higher visual regions important for global shape formation and spatial attention, such as retinotopic areas LO1 and IP0-2 with additional activation in early visual areas, as well as SP. Interestingly, a similar pattern of results was also observed with the addition of interocular stimulus swapping, although the right superior parietal cortex is less activated for stimulus rivalry. Thus, the additional demands of temporal integration in stimulus rivalry did not greatly interact with the demands of spatial integration. Instead, the results were consistent with earlier conclusions that stimulus rivalry is a weaker form of binocular rivalry with shared neural substrates (Buckthought et al. 2015; Petruk et al. 2018). Although we acknowledge the relatively low sample size of this study, we have proceeded cautiously with the aim to offer reproducible conclusions.

### Stimulus Versus Binocular Rivalry

Two previous studies directly compared stimulus and binocular rivalry. Buckthought et al. (2015) showed a pattern of cortical activation that is much weaker for stimulus rivalry as compared to binocular rivalry. Significantly less activity was reported in right parietal cortex, as well as early areas such as V1, V2 and V3 and higher-level visual areas such as VT, MT+ and LOC. It was thus suggested that stimulus rivalry engages early masking mechanisms that are possibly pre-cortical (Baker and Graf 2009; Baker et al. 2007; Brascamp et al. 2013). Consistently, Petruk et al. (2018) used SSVEP to show that binocular and stimulus rivalry shared common neural substrates, with an index of competition co-localized in occipital cortex.

In the current study, we hypothesized that the demands for temporal integration in SR might interact more with the demands for spatial integration than for BR. However, we found little evidence for such a relationship. Except for very small regions, including the anterior cingulate cortex, the BOLD signal evoked for grouped SR was never greater than for grouped BR. Instead, the brain regions activated by grouped SR conditions are similar to those seen for grouped BR (despite the dynamic stimulus changes at the level of retinal input). At the level of cortex, it seems likely that similar binocular representations of competing stimuli can operate within a common mechanistic framework. For example, Pearson and Clifford (2005) juxtaposed pattern, binocular and stimulus rivalry into a single stimulus (a circular disc with three wedge sectors comprised of the three rivalry types). Observers perceived globally coherent dominant patterns which co-varied perceptually across all three rivalry types. Nevertheless, it does not necessarily follow that *all* stages of processing are the same for *all* rivalry types. Such perceptual integration may occur only in higher-tier visual areas, and it is possible that alternations for weaker forms of rivalry are driven or “captured” by the more automatic process of binocular rivalry (Suzuki and Grabowecky 2002; Buckthought et al. 2015). Indeed, studies have demonstrated rivalry to be a process that is distributed across a hierarchy made up of multiple channels and stages (Freeman 2005; Alais and Blake 2005; Lumer 1998; see also Wilson 2003; Bonneh et al. 2001).

### Topography of Lateral Occipital and Parietal Cortex Regions of Interest

Historically, one of the very first fMRI studies of object recognition (Malach et al. 1995) pioneered the concept of comparing objects with scrambled objects and the associated activation in the lateral occipital visual cortex. Subsequently, papers by this group and others relied on the naming convention of the ‘lateral occipital complex’ (LOC) as a term of convenience. This term was useful despite the fact that this subtraction often produces activity in ventral temporal regions of cortex not located on the lateral occipital aspect. Moreover, the use of this localization technique has been widespread, and we refer to this literature in the next section. In this study, we also defined two ROIs called LOR-D and LOR-V; referring respectively to the distinct dorsal and ventral regions recruited by rivalry with interocular grouping. Figure 5 shows these relationships. Although these ROIs are not independently defined, they are useful benchmarks for comparison. It should also be appreciated that the excellent correspondence between the Wang atlas area LO1 and LOR-V, as well as IP0-2 and LOR-D is completely independent. In sum, we aim here to place our newly discovered link between rivalry with interocular grouping to both LO1 and to IP0-2 in the context of other topographical landmarks and naming conventions.

### The Role of Lateral Occipital Cortex in Interocular Grouping

The increased activation for LO regions was evident when comparing grouped and ungrouped binocular rivalry in a direct subtraction as well as with the comparisons to replay. The data also suggest a slight right hemisphere bias of retinotopic area LO1 that is consistent throughout all grouped subtractions. Although much research points to LOC regions as being important for high-level functions such as whole object identification, there is also evidence which suggests that LOC regions are part of a middle vision level of processing, such as shape and contour analysis (Halgren et al. 2003; Ptak et al. 2014). Our grating stimuli proved effective in LO1 recruitment despite the complete lack of semantic content. Rather, the Gestalt rule of good continuation is strongly invoked. This process involves the assignment of borders between different image segments as well as the grouping of similarly patterned elements.

We also suggest that it may be relevant to consider the high responsiveness of LOC regions to stimuli with perceived illusory contours or shapes (Halgren et al. 2003; Mendola et al. 1999; Kourtzi et al. 2003; Kruggel et al. 2001; Stanley and Rubin 2003; Seghier and Vuilleumier, 2006). Illusory contours are comprised of high contrast elements with large intervening gaps (Davis and Driver 1994; Halgren et al. 2003; Senkowski et al. 2005; Seghier and Vuilleumier, 2006). A commonality between illusory contours and perceptual grouping is that both phenomena rely on the construction of global shapes from spatially distributed visual elements. The relatively large receptive fields in this region would be well suited for such integration, perhaps via convergent feedforward input (Majima et al. 2017). It is important to note that retinotopic visual area LO1 has been specifically associated with high sensitivity to illusory contours presented in parafoveal visual field locations (Larsson and Heeger 2006).

In an interesting and partially relevant study, Sutoyo and Srinivasan (2009) observed nonlinear SSVEP responses to grouped stimuli composed of complementary hemifields in either the same, or different eyes. These responses could be a signature of the binding of the parts into a whole percept, and were found for both the suppressed and dominant percepts. We agree with their conclusion that *binocular* competition between percepts contributes to both grouped and ungrouped rivalry. However, their EEG technique did not allow the specific cortical regions selectively recruited for interocular grouping to be clearly identified. Our results suggest that the lateral occipital and parietal regions are likely sites of this binocular mechanism.

### The Role of Parietal Cortex in Interocular Grouping

Parietal cortex has long been associated with binocular rivalry tasks (e.g., Zaretskaya et al. 2013; Lumer and Rees 1999; Blake and Logothetis 2002; Doesburg et al. 2009; de Graaf et al. 2011; Pitts and Britz 2011; Shimono and Niki 2013). However, the increased magnitude of parietal activation we find for the grouped conditions is more than can be explained by aspects related to rivalry only. It is well known that parietal cortex plays a significant role in visuospatial attention and analysis (Shafritz et al. 2002; Robertson et al. 1988; Seymour et al. 2008; Kravitz et al. 2011). Lesions to the inferior parietal lobule specifically yield deficits in spatial perception and visuomotor integration (e.g. Andersen 2011), as well as spatial neglect and simultagnosia in humans (Husain and Nachev 2007; Vallar and Calzolari 2018; Michel and Henaff 2004). Disrupting the focus of spatial attention has been shown to cause binding errors, which results in the combination of wrongly associated features to a given object (Treisman and Schmidt 1982). As is also the case for the LOC region, inferior parietal cortex neurons have large receptive fields with a significant degree of visual input integration (Andersen 2011; Robinson et al. 1978; Yin and Mountcastle 1977). The prominent activation seen here in retinotopic area right IP2 may support the spatial integration of the distinct patches into their respective coherent percepts.

Moreover, it is possible that the spatial attention functions of the parietal cortex are working in tandem with the LO regions in order to effectively solve the binding problems presented in the grouped conditions. There is good evidence that spatial attention influences processing in LOC regions (Kourtzi and Huberle 2005; Martinez et al. 2007; Fesi and Mendola 2013). Martinez et al. (2007) showed that spatial attention to one part of an object could enable the processing of the global object at the level of the LOC. Specifically, both *object-based* and *spatial* attention was associated with intensified negative event related potentials (N1 component) that were co localized with BOLD activations in the LOC. Thus, directing attention to one part of an object can facilitate the processing of the entire object at the level of the LOC (Martinez et al. 2007).

In particular, the spatial attention system of the parietal lobe is likely involved in proper feature binding of *global* forms specifically, by facilitating their processing (Shafritz et al. 2002; Treisman 1998; Zaretskaya et al. 2013; Gilbert et al. 2000; Kourtzi and Huberle 2005; Zhang et al. 2011; Grassi et al. 2016), and in fortifying the representation of whole objects (Martinez et al. 2007). One fMRI study used a non-rivalrous bistable stimulus comprised of moving dots which leads to alternations between local features and a grouped illusory Gestalt percept (Grassi et al. 2016). The parietal IPS was the only region to show activity selective to *only* the global percept (regardless of overall stimulus size). Moreover, when Zaretskaya et al. (2013) studied the same paradigm with TMS, they found that applying a suppressive TMS protocol to the parietal region significantly decreased dominance durations for the grouped percept only. A stronger effect for the right hemisphere is not surprising considering that spatial gestalt encoding has often been shown to be stronger in the right hemisphere (Robertson et al. 1988; Halligan et al. 2003; Huberle and Karnath 2012; Yamaguchi et al. 2000; O'Shea and Corballis 2005). Our finding here of a small trend towards the right hemisphere for grouped perceptual rivalry lends additional support.

In conclusion, these results make an important contribution to the perceptual grouping literature. Although the broad topic of Gestalt perception has been explored behaviorally for decades, our understanding of the relevant neural mechanisms lags behind. Stimulus rivalry (with rapid exchange of stimuli between the eyes), and rivalry with interocular grouping are the best-known prima facie demonstrations that rivalry occurs between *percepts*, not only between eye’s input. Yet, the neural mechanisms of these experiences are very different. The current results reinforce the view that integration for stimulus rivalry occurs as early as the LGN, and hence both eye and percept rivalry occur in similar binocular cortical networks. On the other hand, we interpret our results with recruitment of retinotopic LO and IP regions for rivalry with interocular grouping to specifically reflect a long-range grouping process. Finally, we also suggest that these results provide valuable insight into how the visual system organizes and solves a wide range of complex perceptual problems related to feature binding, pattern analysis, spatial manipulation and object recognition.

## Data Availability

Data will be made available upon reasonable request.
